# Anesthetic Management of Laparoscopic Adrenalectomy for a Patient with Concomitant Pheochromocytoma and Bilateral Carotid Artery Stenosis

**DOI:** 10.1155/2023/2172464

**Published:** 2023-01-07

**Authors:** Kristina L. Michaud, Robert H. Thiele, Katherine T. Forkin

**Affiliations:** Department of Anesthesiology, University of Virginia Health System, P.O. Box 800710, Charlottesville, VA 22908, USA

## Abstract

Symptomatic carotid stenosis and pheochromocytoma both require timely surgical intervention. Following a transient ischemic attack (TIA), a 46-year-old man was diagnosed with bilateral carotid artery stenosis and scheduled for carotid endarterectomy. He was a poor candidate for minimally invasive options due to prior neck radiation. Simultaneously, he began experiencing difficulty with diabetes management and elevated blood pressures and was ultimately diagnosed with pheochromocytoma. This unique situation required coordination to determine the appropriate timing of the two interventions. This case highlights the importance of communication and coordination amongst medical specialists and consideration for anesthetic management of patients with concomitant pheochromocytoma and carotid stenosis.

## 1. Introduction

Resection of pheochromocytoma was historically associated with significant surgical mortality (as high as 50%) [1]. Outcomes have improved dramatically with advancements in preoperative optimization and perioperative management [2, 3]. Profound intraoperative hemodynamic lability is the greatest risk during adrenalectomy for pheochromocytoma resection. Preoperative alpha and beta blockade and use of titratable vasoactive agents intraoperatively are used in an attempt to both maintain adequate perfusion pressure to vital end organs and also avoid significant hypertensive events that could lead to myocardial ischemia or stroke. Potentially provoking events that may trigger a catecholamine surge include noxious stimuli from laryngoscopy, intubation, surgical incision, abdominal insufflation, and manipulation of the tumor [2]. The potential risk of stroke is magnified in a patient with concomitant bilateral common carotid artery stenosis. We present the following medically challenging case involving a patient with both significant bilateral carotid artery stenosis and pheochromocytoma and discuss the multidisciplinary decision making involved.

A written Health Insurance Portability and Accountability Act authorization to use/disclose existing protected health information was obtained for use of this case report and images. This manuscript adheres to the applicable EQUATOR guideline.

## 2. Case Description

A 46-year-old, 86 kg male with past medical history of non-insulin dependent type II diabetes mellitus, hyperlipidemia, hypothyroidism, gout, history of Hodgkin's lymphoma status after chemoradiation therapy (in remission), and recent diagnosis of bilateral carotid artery stenosis and pheochromocytoma presented for laparoscopic adrenalectomy. Six months prior, the patient experienced acute slurred speech, numbness, and left facial twitching while on a fishing trip. Non-contrast head CT scan was obtained and normal. With spontaneous resolution of symptoms, he was diagnosed with a transient ischemic attack (TIA) and discharged home with close follow-up with his primary care provider.

One week later, a non-contrast brain MRI demonstrated a right frontal cortex infarct. CT angiogram of the neck and chest demonstrated 60% stenosis of the left common carotid artery and 60–70% stenosis of the right common carotid artery. Subsequent bilateral carotid artery duplex study showed proximal right common carotid artery with severe stenosis (70–99% occlusion), proximal right internal carotid artery with mild stenosis (1–49% occlusion), proximal left common carotid artery with moderate stenosis (50–69% occlusion), and proximal left internal carotid artery with mild stenosis (1–49% occlusion). His vascular surgeon considered him a poor candidate for minimally invasive intervention of the right and potentially left common carotid arteries due to prior neck radiation, and he was scheduled to undergo carotid endarterectomy.

The patient was also being evaluated for a growing right adrenal mass noted on abdominal CT scan obtained to monitor for recurrence of Hodgkin's lymphoma. CT scan 3 years prior demonstrated a 1.2 cm right adrenal mass, but as he was asymptomatic, a watch, wait, and re-evaluate approach was taken. His most recent CT scan demonstrated that the right adrenal mass had increased in size to 2.2 cm. Around this time, the patient endorsed difficulty in managing his previously well-controlled diabetes, orthostasis, neuropathy in bilateral hands and feet, and occasional muscle cramps. He denied any known family history of endocrine tumors or personal history of hypertension, flushing, palpitations, rapid heartbeat, or headache. Subsequent lab testing was notable for a 24-hour urine normetanephrine of 1400 pg/mL and free normetanephrine of 3.9 pg/mL, and the patient developed significantly elevated blood pressures. He was referred to general surgery for consultation regarding right adrenalectomy for suspected pheochromocytoma. During this time, the patient developed worsening symptoms including consistently elevated blood pressure and heart rate and increasing challenges controlling his blood glucose levels.

The patient's endocrinologist prescribed PO doxazosin for alpha blockade with instructions for home blood pressure and heart rate monitoring. The patient was instructed to increase his dose of doxazosin every 3-4 days until his seated systolic blood pressure was consistently between 90-120 mmHg and his heart rate between 60-70 bpm. He was additionally instructed to maintain a daily sodium intake of greater than 5000 mg and maintain adequate fluid hydration. Once alpha blockade was achieved, he was started on beta blockade with PO atenolol.

On the morning of surgery, the patient presented with well-controlled blood pressure with BP 123/75 mmHg. His anesthesiologist and general surgeon contacted the patient's vascular surgeon to discuss the risks and benefits of proceeding with pheochromocytoma resection prior to carotid intervention. The multidisciplinary team agreed it was appropriate to proceed with adrenalectomy and plan for carotid intervention after postoperative recovery.

The patient was subsequently taken to the operating room for retroperitoneoscopic right adrenalectomy. He was premedicated with 4 mg IV midazolam, and a preinduction arterial line was placed. Induction of general anesthesia was achieved with lidocaine and propofol with rocuronium for muscle paralysis, and intubation was performed successfully on the first attempt with direct laryngoscopy. Bilateral cerebral oximetry was used to monitor cerebral oxygen saturation throughout the case. Sevoflurane was used for maintenance of anesthesia. Ketamine boluses were administered for anti-nociception. Dexamethasone and ondansetron were administered for postoperative nausea and vomiting (PONV) prophylaxis. A total of 10 units of insulin were administered to treat blood glucose elevations in the low 300 s. Intraoperative hypotension following resection of the tumor was treated with volume resuscitation with crystalloid and norepinephrine and vasopressin infusions and boluses. Cerebral oximetry remained within 20% of baseline throughout. The operation was performed without complication.

Following the operation, the patient was extubated and brought to PACU in stable condition. Postoperative pain was well controlled with oxycodone and acetaminophen. Postoperative course was relatively uneventful with the exception of persistent hyperglycemia requiring consultation of the inpatient endocrinology service, while in the hospital, the patient's blood pressure medications were held and his blood pressure remained well controlled. On postoperative day 1, the patient was alert and oriented, vitals were stable, and pain was well controlled, ambulating with very little assistance, tolerating a regular diet, and voiding independently. He was sent home with instructions to follow-up with endocrinology and general surgery and to continue to closely monitor his blood pressure.

## 3. Discussion

**Figure 1:**
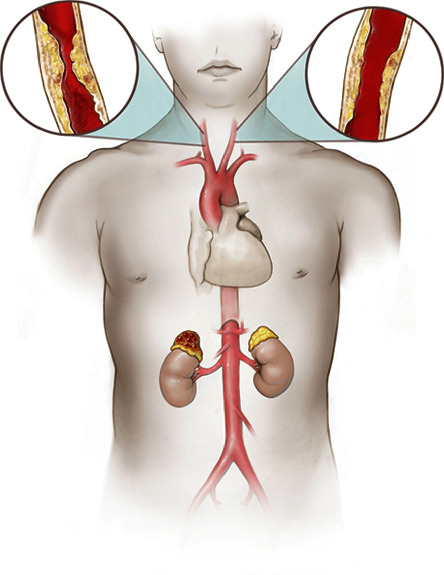
Representation of our patient's pathology—right pheochromocytoma with bilateral carotid artery stenosis with the right common carotid artery with severe stenosis (70–99% occlusion) and left common carotid artery with moderate stenosis (50–69% occlusion).

Our patient was diagnosed with pheochromocytoma and bilateral carotid artery stenosis (Figure 1), the latter of which was likely caused by prior neck radiation for Hodgkin's lymphoma, both requiring timely intervention. Adrenalectomy and carotid endarterectomy both carry significant potential risks and complications. Each procedure is time-sensitive, with delays in intervention potentially increasing morbidity and mortality.

Pheochromocytoma is a rare neuroendocrine tumor of the adrenal medulla that produces, stores, and releases catecholamines (epinephrine, norepinephrine, and/or dopamine) into systemic circulation causing a variety of signs and symptoms including diaphoresis, headache, poorly controlled hypertension, palpitations, flushing, pallor, and anxiety (Table 1). Triggers of catecholamine release include stress, certain medications, pain, manipulation of the abdomen or tumor, and positional changes. Adrenalectomy for pheochromocytoma once portended up to 50% mortality rate, although this has decreased dramatically over time [2, 3]. The mainstay of preoperative management involves antihypertensive treatment first with alpha blockade followed by beta blockade and/or calcium channel blockade [4]. It is also important to evaluate patients preoperatively for cardiovascular sequelae from their high circulating amount of catecholamines such as tachyarrhythmias, vasoconstriction of the coronary arteries, arterial stiffness, and acute cardiomyopathies (including dilated, hypertrophic, or Takotsubo cardiomyopathy) [2, 5, 6]. Additionally, preoperative therapy should include a high-sodium diet and adequate fluid hydration to counteract the catecholamine-induced blood volume contraction [5]. These preoperative therapies help mitigate perioperative hemodynamic instability. Common perioperative stimuli that may provoke hypertensive crises during adrenalectomy prior to tumor resection include laryngoscopy, endotracheal intubation, abdominal insufflation, surgical incision, and manipulation of the tumor. It is important to recognize that these stimuli can provoke hypertensive crises in patients with pheochromocytoma (diagnosed or undiagnosed) undergoing any procedure. Following tumor resection, significant hypotension may occur due to the reduction in catecholamines, often resolving within 24 hours [2]. In our patient with symptomatic bilateral carotid artery stenosis, maintaining adequate cerebral perfusion pressure was particularly critical.

When considering the management of symptomatic carotid stenosis, carotid revascularization is recommended as the most optimal treatment in stroke prevention. The recommended timing of poststroke carotid endarterectomy is generally within 14 days after the onset of symptoms [7–9]. There is high risk (9–12%) of stroke recurrence within the subsequent 6 weeks without intervention [10]. As our patient was concomitantly undergoing workup for pheochromocytoma, it was not appropriate to proceed with carotid intervention before that workup was complete.

Undergoing carotid endarterectomy in the setting of a non-resected pheochromocytoma would entail significant potential risk. The end goal of medical optimization with alpha and beta blockade for pheochromocytoma is orthostatic hypotension; for a patient with significant carotid artery stenosis, hypotension increases the risk of cerebral ischemia. Thus, the patient ideally would not be continued on alpha and beta blockade any longer than necessary. Furthermore, a potential complication following carotid endarterectomy includes postoperative hypotension due to increased sensitivity of the carotid baroreceptors following excision of the plaque. In a patient who already is receiving alpha and beta blockade for medical management of their pheochromocytoma, this phenomenon would likely be exacerbated, thereby placing the patient at high risk of post-endarterectomy stroke. However, proceeding with adrenalectomy for pheochromocytoma in the presence of moderate to severe bilateral carotid artery stenosis is not without risk. It was ultimately decided after multidisciplinary (anesthesiologist, surgeon, and neurologist) planning and consideration that proceeding with pheochromocytoma resection with adequate alpha and beta blockade and hydration preoperatively would be more appropriate to perform first. After surgical recovery with improved hemodynamic stability, the patient would be able to proceed with carotid intervention with his vascular surgeon.

In summary, patients with pheochromocytoma and symptomatic carotid artery stenosis should proceed first with adrenalectomy with thoughtful perioperative management. Intraoperative use of cerebral oximetry by near-infrared spectroscopy during the adrenalectomy allows for additional monitoring to try to identify any episodes of cerebral hypoperfusion that could potentially be quickly resolved with vasopressor administration or fluid resuscitation [11]. Carotid intimal media thickness has been shown to be increased in patients with pheochromocytoma due to vascular remodeling from circulating catecholamines [12]. Thus, concomitant pathologies may occur with some frequency. This case highlights the importance of multidisciplinary discussion and thoughtful planning for complex patients to optimize perioperative management and postoperative outcomes.

## Figures and Tables

**Table 1:** Most common signs and symptoms of pheochromocytoma.

Signs	Symptoms
Paroxysmal or sustained hypertension	Headache
Orthostatic hypotension	Palpitations
Tachycardia or reflex bradycardia	Anxiety, panic
Excessive sweating	Tremulousness
Pallor	Diaphoresis
Hyperglycemia	Dyspnea

Weight loss	Weakness, fatigue
	Heat intolerance
	Abdominal pain
	Nausea, vomiting
	Visual disturbances

## Data Availability

All relevant data are included in this article, and any other data used to support the conclusions of this case report are restricted by HIPPA in order to protect patient privacy.
